# Melatonin in cryopreservation media improves transplantation efficiency of frozen–thawed spermatogonial stem cells into testes of azoospermic mice

**DOI:** 10.1186/s13287-022-03029-1

**Published:** 2022-07-26

**Authors:** Shokoofeh Kazemzadeh, Shahram Mohammadpour, Soheila Madadi, Azar Babakhani, Maryam Shabani, Maryam Khanehzad

**Affiliations:** 1 Department of Anatomy, School of Medicine, Tehran University of Medical Sciences, Tehran, Iran; 2 Department of Anatomy, School of Medicine, Ilam University of Medical Sciences, Ilam, Iran; 3 Biotechnology and Medicinal Plants Research Center, Ilam University of Medical Sciences, Ilam, Iran; 4 Department of Anatomy, School of Medicine, Arak University of Medical Sciences, Arak, Iran; 5 Department of Clinical Biochemistry, School of Medicine, Tehran University of Medical Sciences, Tehran, Iran; 6 Department of Anatomical Sciences and Molecular Biology, School of Medicine, Isfahan University of Medical Sciences, Isfahan, Iran

**Keywords:** Melatonin, Spermatogonial stem cells, Cryopreservation, Transplantation

## Abstract

**Background:**

Cryostorage of spermatogonial stem cells (SSCs) is an appropriate procedure for long-term storage of SSCs for fertility preservation. However, it causes damage to cellular structures through overproduction of ROS and oxidative stress. In this study, we examined the protective effect of melatonin as a potent antioxidant in the basic freezing medium to establish an optimal cryopreservation method for SSCs.

**Methods:**

SSCs were obtained from the testes of neonatal male mice aged 3–6 days. Then, 100 μM melatonin was added to the basic freezing medium containing DMSO for cryopreservation of SSCs. Viability, apoptosis-related markers (BAX and BCL2), and intracellular ROS generation level were measured in frozen–thawed SSCs before transplantation using the MTT assay, immunocytochemistry, and flow cytometry, respectively. In addition, Western blotting and immunofluorescence were used to evaluate the expression of proliferation (PLZF and GFRα1) and differentiation (Stra8 and SCP3) proteins in frozen–thawed SSCs after transplantation into recipient testes.

**Results:**

The data showed that adding melatonin to the cryopreservation medium markedly increased the viability and reduced intracellular ROS generation and apoptosis (by decreasing BAX and increasing BCL2) in the frozen–thawed SSCs (*p* < 0.05). The expression levels of proliferation (PLZF and GFRα1) and differentiation (Stra8 and SCP3) proteins and resumption of spermatogenesis from frozen–thawed SSCs followed the same pattern after transplantation.

**Conclusions:**

The results of this study revealed that adding melatonin as an antioxidant to the cryopreservation medium containing DMSO could be a promising strategy for cryopreservation of SSCs to maintain fertility in prepubertal male children who suffer from cancer.

**Supplementary Information:**

The online version contains supplementary material available at 10.1186/s13287-022-03029-1.

## Introduction

Spermatogonial stem cells (SSCs) are germ cells located at the base of the seminiferous tubules, which are capable of self-renewal and differentiation and are important for maintaining the complex process of sperm production and male fertility [1, 2]. Radiation therapy and chemotherapy for cancer treatment can cause defects in the germinal epithelium and spermatogenesis leading to infertility. Busulfan is frequently used as a chemotherapy drug and often leads to defects in spermatogenesis and infertility. Irreversible and permanent fertility damage caused by anticancer drugs affects the patients’ quality of life in the future. Due to this side effect of chemotherapy, it is possible to maintain fertility in adult men suffering from cancer by using sperm cryopreservation; however, prepubertal boys lack spermatogenesis and their semen is not available for cryopreservation and long-term storage. Therefore, isolation, culture, and freezing of SSCs for long-term storage may be a suitable method to maintain and restore future fertility in these patients before using anticancer drugs [3, 4].

Cryopreservation is performed at subzero temperatures for long-term storage and protection of living cells and tissues. Despite the advantages of this method, cryopreservation is associated with a wide range of damage, including the formation of intracellular and extracellular ice crystals and osmotic stress, which reduces the efficiency of living cells and tissues after the freezing–thawing process. To prevent damage at very low temperatures, the researchers add chemicals known as cryoprotectants to the cells [5]. Cryoprotectant agents (CPAs) are divided into permeable and impermeable groups based on the material characteristics. Permeable CPAs (small molecules) such as dimethyl sulfoxide (DMSO) and ethylene glycol (EG) are able to form hydrogen bonds with water molecules after penetration into the cell membranes and lower the freezing point and prevent the formation of intra- and extracellular ice crystals and injuries during cryopreservation. Impermeable CPAs such as trehalose and polyvinyl pyrrolidone are not able to penetrate into cell membranes due to their high molecular weight. These types of cryoprotectants can cause changes in the physical state of the extracellular compartment of the cryopreservation medium, leading to the formation of sheaths around the cells and prevention of cellular dehydration during cryopreservation [6, 7]. In addition, cell cryopreservation is associated with production of super-physiological levels of reactive oxygen species (ROS), which can contribute to DNA damage, lipid peroxidation, apoptosis and reduced cell viability during the freezing and thawing process [8]. Although permeable and impermeable CPAs have two different mechanisms for reducing injury during cryopreservation, they fail in disposing ROS and reducing oxidative damage to cells. Therefore, adding antioxidants to the basic freezing medium, as ROS scavengers, can partially or completely prevent the destructive effects of cryoinjury in cryopreserved cells through decreasing oxidative stress and ROS production, transmuting ROS into harmless materials, and increasing the activity of antioxidant enzymes [9]. Recent studies have shown that adding antioxidant supplements such as melatonin [10], resveratrol [11], lycopene [12], α-tocopherol, and ascorbic acid [13] to CPAs can prevent oxidative stress and injury induced by the production of excess ROS during cryopreservation. Hence, a combination of antioxidants and cryoprotectant agents (CPAs) in the cryopreservation media may reduce osmotic and mechanical injuries caused by intracellular and extracellular ice crystals, DNA damage, lipid peroxidation, and protein oxidation and promote the efficiency of cryopreservation in the future [9].

In mammals, melatonin is secreted as the basic hormone of the pineal gland. Its antioxidant and ROS-scavenging role are well established [14]. Several studies have shown that melatonin is effective in proliferation and self-renewal of SSCs and spermatogenesis and improves the testicular and reproductive system function in men [15–19]. The antioxidant and anti-inflammatory effects of melatonin have been observed in the treatment of male reproductive system injuries. In addition, it has been shown that the melatonin acts as a cryoprotectant in the freezing media containing sperm and positively affects male fertility preservation [20–22]. Previous research has shown that adding melatonin to the culture medium increases the efficiency and performance of SSCs isolated from mice [15, 16]. Cryopreservation of SSCs can provide appropriate options such as SSCs transplantation and in vitro spermatogenesis for prepubertal boys and adult male after cancer treatment [23]. Therefore, cryopreservation of SSCs can be a vital step for their long-term storage and maintenance of male fertility [24].

In order to improve the recovery of frozen–thawed SSCs in the cryopreservation method, we investigated the protective effect of melatonin in the cryopreservation medium on the proliferation and differentiation of post-thawed SSCs after transplantation into the testes of azoospermic mice.

## Materials and methods

### Animal grouping and study design

In this research, 58 adult male NMRI mice (6 weeks old) weighing 28–30 g were obtained from the Animal Lab of the School of Pharmacology of Tehran University of Medical Sciences. The animals were housed at standard temperature and humidity with a 12/12 h light–dark cycle, free access to water and food, and pathogen-free conditions. All experiments related to the use of animals in this study were approved by the Ethics Committees of Tehran University of Medical Science (IR.TUMS.MEDICINE.REC.1397.213).

Adult male NMRI mice (6 weeks old) were randomly divided into four groups as control group (mice without any injections and transplantations); busulfan group (intraperitoneal injection of busulfan to induce azoospermia model); transplantation group 1 (freezing SSCs: transplantation of frozen–thawed SSCs in the basic freezing medium into testes of azoospermic mice); transplantation group 2 (freezing SSCs + melatonin: transplantation of frozen–thawed SSCs in the basic freezing medium containing melatonin into testes of azoospermic mice).

Fifty-eight mice were initially kept in control (*n* = 13), busulfan (*n* = 13), transplantation 1 (*n* = 16) and transplantation 2 (*n* = 16). Seven weeks after busulfan injection, 12 mice (*n* = 3 per group) were killed to confirm the azoospermia model. Two weeks after transplantation of the thawed–thawed SSCs, six mice in transplantation groups 1 and 2 (*n* = 3 per group) were killed to confirm the presence of transplanted SSCs in the seminiferous tubules. Finally, forty mice (*n* = 10 per group) were killed 8 weeks after transplantation and then their testes were removed for further experiments.

### Spermatogonial cells isolation, purification, and culture

Testes were obtained from 3- to 6-day-old male NMRI mice (*n* = 30), and the testicular cells were isolated according to a two-step enzymatic digestion protocol [25]. The collected testicles were transferred to a dish under sterile conditions. After removing the tunica albuginea from the outer surface of the testicles, the testicular tissue was minced into small pieces. In the first stage, small minced samples were suspended in an enzyme digestion medium containing Dulbecco’s modified Eagle medium (DMEM) supplemented with 1 mg/mL type IV collagenase (Gibco, CA), 1 mg/mL hyaluronidase (Sigma‐Aldrich) and 5 μg/mL DNase (Sigma‐Aldrich). Finally, the enzymatic digestion medium containing testicular cells was transferred to a 5% CO_2_ incubator at 37 °C for 20 min. The samples were then pipetted every 5 min to disintegrate the seminiferous tubules and form a cell suspension. At the end of the first step, the cell suspension was centrifuged at 1500 g for 5 min. The second stage was performed with the same method and the enzymes used in the first stage, and the cell suspension was kept in the incubator for 15 min and then the final centrifugation was performed for 5 min (1500 g). The formed cell pellet was washed with PBS, and the isolated cells were purified using the differential platting technique. Floating non-adherent cells were collected in the culture dish after purification using the differential platting method. To determine the percentage of purified SSCs, specific markers of PLZF, Thy1 and α6-integrin in germ cells were assessed using flow cytometry. For this purpose, 10 μl of primary antibodies including PLZF (1:100; ab104854; Abcam, Cambridge), Thy-1 (1:100; ABIN728038; Antibodies-Online) and α-6 integrin (1:100; ab235905; Abcam, USA) was added to the purified testicular cells (1 h at room temperature). After washing the cells with PBS, 10 μl of secondary antibody (goat anti‐rabbit IgG H&L; ab6717, Abcam) conjugated with fluorescein isothiocyanate (FITC) was added to the cells and incubated at 4 °C for 1 hour. Testicular cells that were not treated with antibodies were considered as negative control. Purified SSCs were cultured in a basic culture medium (alpha-MEM) supplemented with 0.1 mM β‐mercaptoethanol (Sigma‐Aldrich), 10 ng/Ml leukemia inhibitory factor (LIF, Sigma‐Aldrich),100 μg/mL streptomycin (Sigma, Germany), 10% fetal bovine serum (FBS, Life Technologies), 1% non‐essential amino acids (Gibco, Invitrogen), 100 U/mL penicillin (Sigma‐Aldrich),10 ng/mL basic fibroblast growth factor (bFGF, PeproTech, Rocky Hill, NJ) and 10 ng/mL glial cell line-derived neurotrophic factor (GDNF; Sigma-Aldrich). The dishes containing the cultured cells were transferred to an incubator with 5% CO_2_ at 37 °C for 2 weeks. Then, the culture medium was replaced with fresh medium every 3 days during the culture period.

### Cryopreservation and thawing procedure

Cryopreservation of SSCs was performed 2 weeks after cell culture. The basic freezing medium used to freeze cells included MEM-α (Sigma, Germany), 10% fetal bovine serum (FBS; Sigma, Germany) and dimethyl sulfoxide (DMSO; 1.4 M). Two freezing solutions were used to freeze the cells. The first freezing solution in the control group contained basic freezing medium without additional supplements, and the second freezing solution used in the experimental groups contained the basic freezing medium supplemented with melatonin at doses of 50, 100 and 150 μM. Freezing solutions were slowly added to 1.8-ML cryovials containing cell suspension. The cryovials were placed in a Nalgene freezing container and stored overnight at − 80 °C [26]. After freezing overnight at − 80 °C, cryovials containing frozen cells were transferred to liquid nitrogen for at least a month. Cryovials containing frozen cells were removed from liquid nitrogen after freezing for 1 month, and the frozen cells were incubated in a 37 °C water bath (2 min). Then, the thawed cells were diluted with a medium containing MEM alpha and 10% FBS in a dropwise manner and centrifuged (1200 × *g*, 5 min) [27]. After discarding the supernatant, the cell pellet formed was evaluated to compare the performance of cryopreservation medium between the control (basic freezing medium) and treatment (basic freezing medium + melatonin) using MTT assay (viability), flow cytometry (ROS-level measurement) and immunocytochemistry (apoptotic markers) techniques.

### Pre-transplantation evaluations

#### Cell viability

A comparison of cell viability between control (basic freezing medium) and treatment (basic freezing media + melatonin) groups after thawing was made using methyl thiazol tetrazolium assay (MTT; Sigma‐Aldrich). In all groups, 400 μl MEM and 40 μl MTT were added to each well of a 96-well plate containing 400 cells and the plates were incubated at 37 °C for 4 h. After incubation, the supernatant was discarded and 400 μl DMSO was added to each well. Then, the cells were incubated at room temperature (30 min) to dissolve formazan precipitate formed in DMSO and the optical density (OD) was measured at 540 nm using an automatic microplate reader.

#### Intracellular ROS measurement

The intracellular ROS level in SSCs was measured in the freezing and fresh groups using 2’,7’-dichlorohydrofluorescein diacetate (H2DCFDA) probe, which is the most common method for measuring hydrogen peroxide (H2O2) in living cells. After adding DCFDA (10 μl, Sigma, Germany) to the cells and incubating at 37 °C (25 min), DCFDA crosses the cell membrane and is oxidized to 2′,7′-dichlorofluorescein (DCF) in the presence of ROS (predominantly H2O2). Then, the value of the fluorescent in the DCF formed as a high fluorescent product was measured using flow cytometry between 500 and 530 nm [28].

#### Immunocytochemistry

Bax and Bcl2 proteins, as markers related to apoptosis, were detected in SSCs in different groups using immunocytochemistry. The SSCs were fixed in freshly prepared paraformaldehyde 4% for 10 min. To facilitate the penetration of the antibodies, the SSCs were permeabilized with adding 0.1% Triton X‐100 (Sigma‐Aldrich) for 10 minutes; then, the cells were washed with PBS containing 1% bovine serum albumin (BSA). After blocking the sites of nonspecific antigens using 10% goat serum (Sigma-Aldrich) for 1 hour, the slides were incubated with primary antibodies against BAX (1:500, Santa Cruz, USA) and BCL2 (1:600, Biorbyt, UK) for 24 h. Following washing with PBS, FITC Goat Anti-Rabbit IgG (H&L) secondary antibody (1:1000, Abcam, USA) was then added to the slides for 2 hours at room temperature. The cell nucleus was stained with DAPI (1/1500). The immunostained slides were observed under a fluorescence microscope (Olympus LX71, Japan). Then, the stained areas were measured using the ImageJ software.

#### Transplantation procedure and optimization of mouse model for transplantation

To optimize the mouse model and deplete endogenous spermatogenesis in the tubules for transplantation, busulfan (Sigma-Aldrich) was injected to mice intraperitoneally at a single dose of 40 mg/kg. Four weeks after busulfan injection, cadmium chloride (CdCl2, 2 mg/kg) was injected into mice intraperitoneally to ensure lack of function in Sertoli cells and establish an irreversible azoospermia model [29]. Finally, 7 weeks after busulfan injection, the testes of the mice were evaluated using hematoxylin and eosin (H&E) staining to confirm the removal of SSCs from the tubules to induce an azoospermia model. Following optimization of the model, the thawed cells were labeled with the fluorescent dye CM-Dil (Molecular Probes™, USA) based on the manufacturer's protocol and injected into the efferent duct in the recipient testes [30]. For this purpose, the mice were first anesthetized by intraperitoneal injection of 100 mg/kg ketamine and 20 mg/kg xylazine. Then, about 10 μl of the cell suspension containing 10^5^ cells (SSCs labeled with DiI) was injected into the efferent duct in the recipient testis using a needle microinjection under a stereomicroscope [31]. Trypan blue was added to the injection medium containing the cells to track and visualize the tubules. The seminiferous tubules in each recipient testis were approximately 90% filled after transplantation. Two weeks after transplantation, the homing and presence of frozen–thawed SSCs injected into seminiferous tubules (confirmation of transplanted cells) were assessed using immunofluorescence staining. The mice were killed by cervical dislocation 2 months after transplantation of post-thaw SSCs, and the testes were then removed. Finally, proliferation (PLZF, GFRA1) and differentiation (SCP3, Stra8) markers were evaluated using immunofluorescence and western blot techniques.

### Post-transplantation evaluations

#### Immunofluorescence staining

Two months after transplantation, sections prepared from the testicular tissue were deparaffinized and dehydrated; then, the sections were boiled in buffer citrate at 100 °C (20 min) for antigen retrieval. In order to block nonspecific antigens and ensure permeabilization, the sections were exposed to 0.1% bovine serum albumin (BSA, Sigma-Aldrich, USA) in 0.1% triton X-100 for 1 h. Then, the sections were incubated at 4 °C overnight with the primary antibodies against the following markers (PLZF, SCP3): anti-PLZF (1:500, Santa Cruz, USA) and anti-SCP3 (1:400, Abcam, USA). Next, the sections on the slides were washed with PBS, and goat anti-rabbit secondary antibody conjugated with FITC (1:400, Abcam, USA) was added to the slides and maintained at room temperature for 2 h. Tissue sections of the testis were stained with DAPI to detect the cell nucleus and then observed under a fluorescence microscope (Olympus LX71, Japan) equipped with a camera.

#### Western blot

Proteins were extracted from testicular tissue specimens using radioimmunoprecipitation assay buffer (RIPA, Abcam, Germany) supplemented with protease and phosphatase inhibitors and centrifuged. After determining the concentration of proteins using BCA Protein Assay Kit (Abcam, USA), an equal amount of the extracted protein in each sample was separated using SDS-PAGE and transferred to the nitrocellulose membrane. Following blockage of membranes in 5% non‐fat dry milk for 2 h at room temperature, the membranes were incubated with primary antibodies against Stra8 (1:1000, Abcam, USA) and Gfra1 (1:1000, MyBioSource, Inc.) at 4 °C overnight. Then, the membranes were washed three times with Tris buffered saline with 0.1%Tween 20 (TBS-T, Abcam, Germany) and incubated with horse radish peroxidase (HRP)-conjugated goat anti-rabbit IgG secondary antibody (1:2000, Abcam, USA) for 1 h at room temperature. The expression rate of Stra8 and GFRA1 proteins was assessed using enhanced chemiluminescence. Finally, the intensity of the bands was normalized to GAPDH and analyzed using the ImageJ software.

#### Evaluation of histological by H&E staining

Two months after transplantation, the testes of the recipient mice were immersed in Bouin fixative solution for 48 h. Then, the fixed samples were embedded in paraffin. Subsequently, 5-μm-thick sections were prepared from the samples for the routine stages of histological processing and hematoxylin and eosin (H&E) staining procedure. Finally, 50 sections were prepared from each testicular sample and 100 seminiferous tubules (from each section) were randomly selected for spermatogenesis evaluation [32].

### Statistical analysis

The data were analyzed using one-way ANOVA followed by post hoc Tukey’s test for comparison between different groups (GraphPad Software version 6.01, CA, USA). The results are presented as mean ± standard error of the mean (M ± SEM). The level of significance was set at *p* ≤ 0.05.

## Results

### Purification rate of SSCs by flow cytometry

**Fig. 1:**
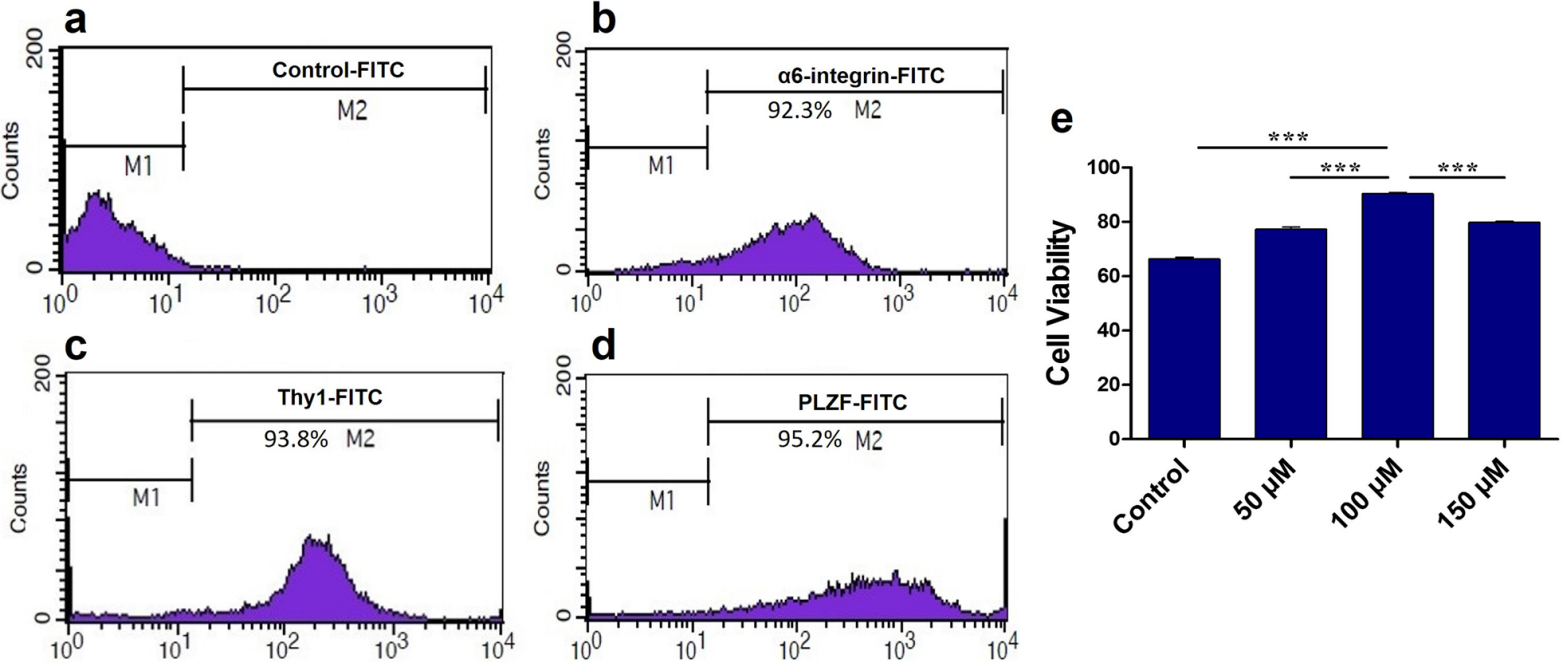
Flow cytometry to determine the purity percentage of isolated SSCs after two-step enzymatic digestion with α6-integrin, Thy1 and PLZF markers. M1: marker negative cells, M2: marker positive cells. **a** control; **b** α6-integrin positive; **c** Thy1 positive; **d** PLZF positive. **e** MTT analysis for assessment viability of frozen–thawed SSCs with different concentrations of melatonin. The results are presented as mean ± SD. ***p < 0.001

To identify the purity rate of SSCs, the expression of PLZF, Thy-1 and Thy-6 integrin-specific markers in testicular cells was evaluated after two-stage enzymatic digestion and differential plating by flow cytometry. The results of flow cytometry showed the high expression rates of specific markers (PLZF, Thy-1 and α-6 integrin) of SSCs. The expression percentage of PLZF, Thy1 and α-6 integrin in SSCs was 95.2%, 93.8% and 92.3%, respectively (Fig. 1a–d).

### Effect of melatonin on viability of SSCs

Four weeks after freezing SSCs, the survival rate of thawed cells in the treatment (Mel + basic freezing media) and control (basic freezing media) groups was assessed using MTT assay. Administration of melatonin at three different concentrations (50, 100 and 150 μM) significantly improved cell survival in the treatment groups compared to the control group (*p* < 0.001). The results show that the cell viability rate in the freezing + Mel group at a concentration of 100 μM (90.4 ± 0.75%) had a significant increase compared to the freezing + Mel groups at concentrations of 50 (77.30 ± 1.44%) and 150 (79.80 ± 0.7%) μM and had the highest survival compared to other groups (*p* < 0.001). However, the lowest survival was seen in the control group (66.33 ± 1.15%). Therefore, subsequent experiments were done by adding 100 μM melatonin to the basic freezing medium (Fig. 1e).

### Effect of melatonin on ROS generation

**Fig. 2:**
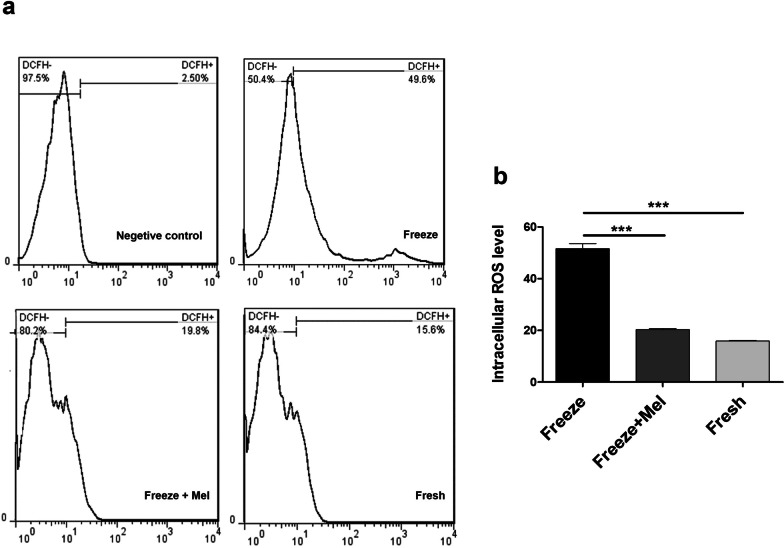
Flow cytometry analysis for intracellular ROS assay in SSCs. **a** Graphs show the percentage of positive ROS cells in different groups SSCs. **b** ROS generation results in freeze, freeze + Mel (100 μM) and fresh groups, analyzed by flow cytometry. The results are presented as mean ± SD. ***p < 0.001

To evaluate the protective effect of melatonin on frozen–thawed SSCs, the level of intracellular ROS production in the cells was measured using flow cytometry in three groups: freeze, freeze + Mel, and fresh. As shown in Fig. 2, intracellular ROS production in the freeze group (51.60 ± 2.82%) increased significantly compared to the freeze + Mel (20.25 ± 0.63%) and fresh (15.85 ± 0.35%) group. As expected, ROS production was significantly reduced in the freeze + Mel group (20.25 ± 0.63%) compared to the freeze group (51.60 ± 2.82%), while there was no significant difference between the freeze + Mel and fresh groups (*p* > 0.05) (Fig. 2). The results of this experiment showed that adding melatonin to the freezing medium reduced the level of intracellular ROS production during the freezing–thawing process.

### Immunocytochemistry results for apoptosis-related markers in SSCs

**Fig. 3:**
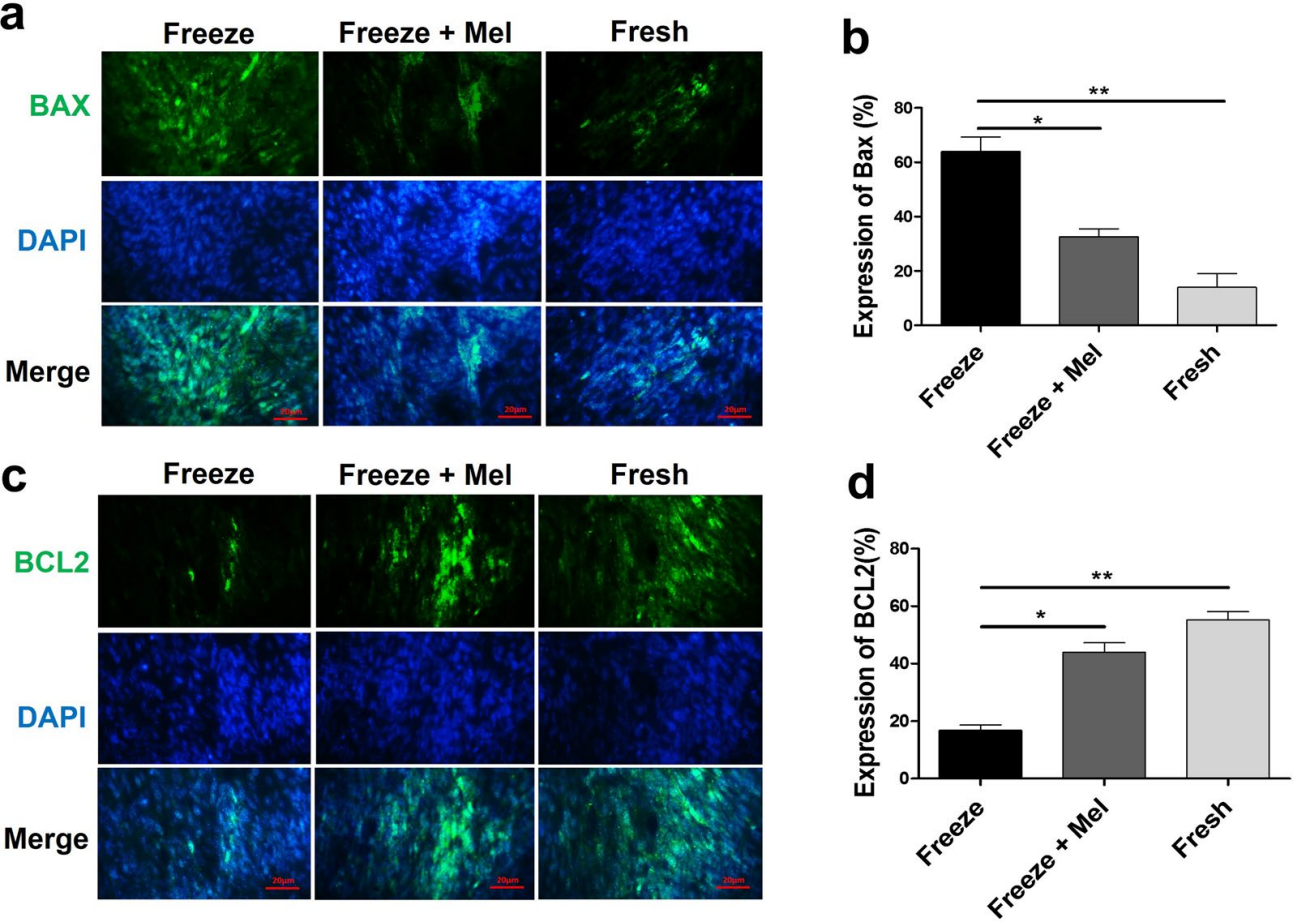
Immunocytochemistry was used for evaluation BAX and BCL2 markers (green) in different groups (freeze, freeze + Mel and fresh) of SSCs before transplantation into recipient testes. The nuclei were stained with DAPI dye (blue). Scale bars = 20 μm (**a**, **c**). The graph depicts the quantitative analysis for the immunocytochemistry results (**b**, **d**). The results are presented as mean ± SD. Significance is indicated by **p < 0.01; *p < 0.05

Expression of apoptosis-related proteins (BAX and BCL2) in frozen–thawed SSCs was evaluated using immunocytochemistry. According to images, adding melatonin to the basic freezing medium reduced and increased the expression of BAX (Fig. 3a) and BCL2 (Fig. 3c) proteins, respectively. The results of quantitative immunocytochemistry analysis revealed that melatonin significantly decreased the expression level of BAX pro-apoptotic protein in the freeze + Mel group compared to the freeze group (32.55 ± 4.17 vs 63.95 ± 7.56%) (*p* < 0.05) (Fig. 3b). Furthermore, the BCL2 protein expression was significantly higher in the freeze + Mel group compared to the freeze group (44.05 ± 4.59 vs 16.85 ± 2.473%) (*p* < 0.05) (Fig. 3d) and there was no significant difference in the expression BAX and BCL2 proteins between fresh and freeze + Mel groups (*p* > 0.05). These findings suggest that melatonin may play a protective role on SSCs in the freezing medium during the freeze–thaw process by reducing the expression of BAX protein and increasing the expression of BCL2 protein (apoptosis reduction).

### Optimization of mouse model for transplantation

**Fig. 4:**
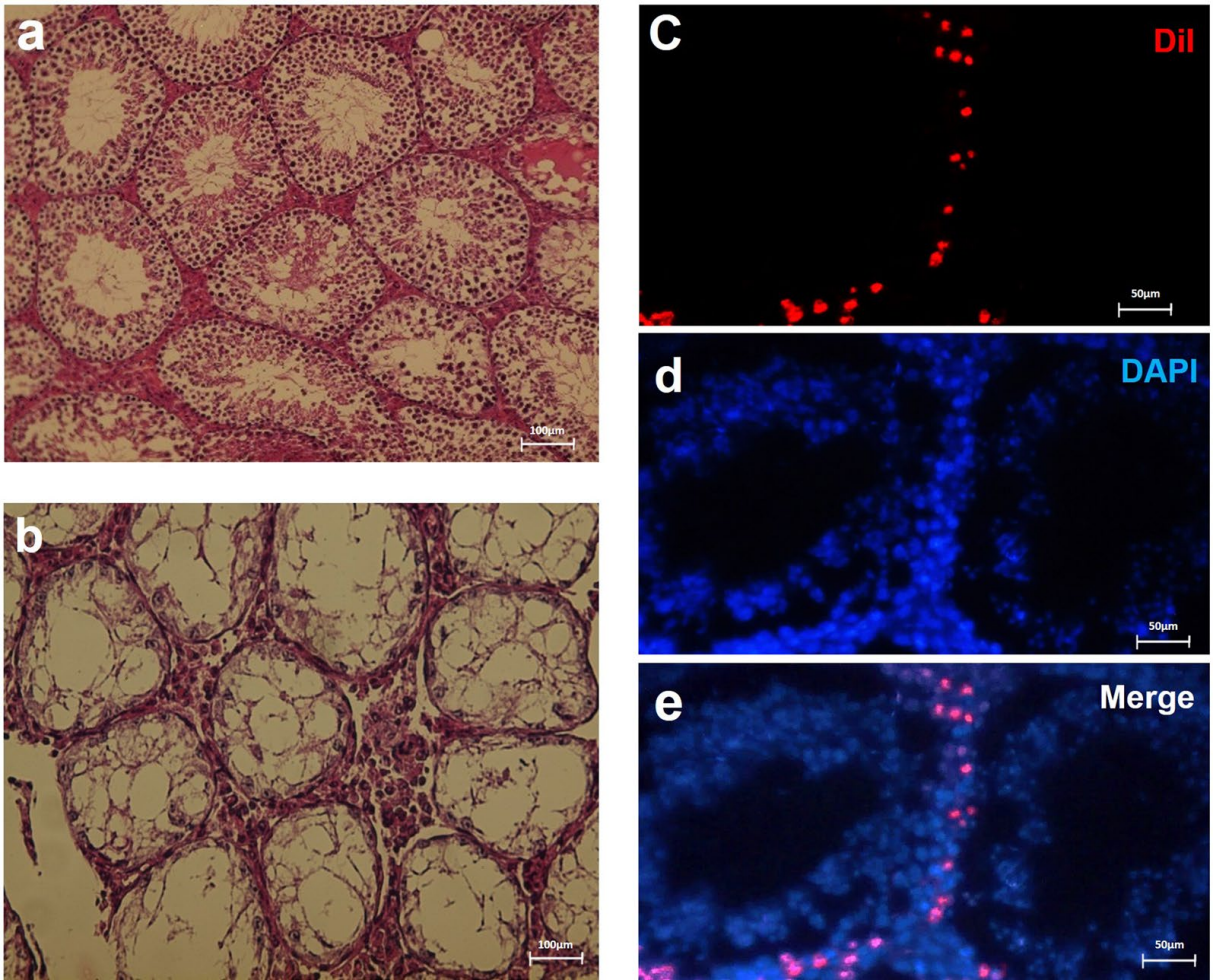
Optimization of the mouse model for transplantation and transplantation confirmation of post-thawed SSCs. In the control group, the seminiferous tubules are completely filled showing all maturation stages of the germinal epithelium from spermatogonia to spermatozoa (**a**). In the busulfan group, most tubules are empty and the germinal epithelium in the seminiferous tubules is severely disrupted (**b**) (scale bar = 100 m). Two weeks after transplantation, homing of DiI-labeled SSCs (DiI.^+^ germ cells) in the basement membrane of seminiferous tubules as confirmed by immunofluorescence staining of recipient testes indicated successful transplantation (**c**). The blue color in same section of the testicular tissue indicated nuclei stained by DAPI (**d**). Merged images are reported in panel (**e**) (scale bars: 50 μm)

To confirm the azoospermia model, tissue sections prepared from the testes of mice in control and busulfan groups (7 weeks after injection) were evaluated using hematoxylin and eosin (H&E) staining. Histological findings of the control group showed that the germinal epithelium in the seminiferous tubules had SSCs and different stages of spermatogenesis and the seminiferous tubules were entirely filled with germ cells (Fig. 4a). However, in the busulfan group, the germinal epithelium was strongly disrupted and the absence of SSCs and spermatogenesis stages was seen in most tubules (Fig. 4b). To confirm transplantation, transplanted SSCs to the testis were observed under a fluorescence microscope. SSCs labeled with red fluorescent light (DiI-labeled SSCs) were considered as the origin of the transplanted cells. Fourteen days after transplantation, the presence of fluorescent-labeled SSCs was detected at the base of the seminiferous tubules, indicating homing and localization of the transplanted cells in the depleted tubules (Fig. 4e).

### Immunofluorescent findings for expression of PLZF and SCP3 proteins in SSCs

**Fig. 5:**
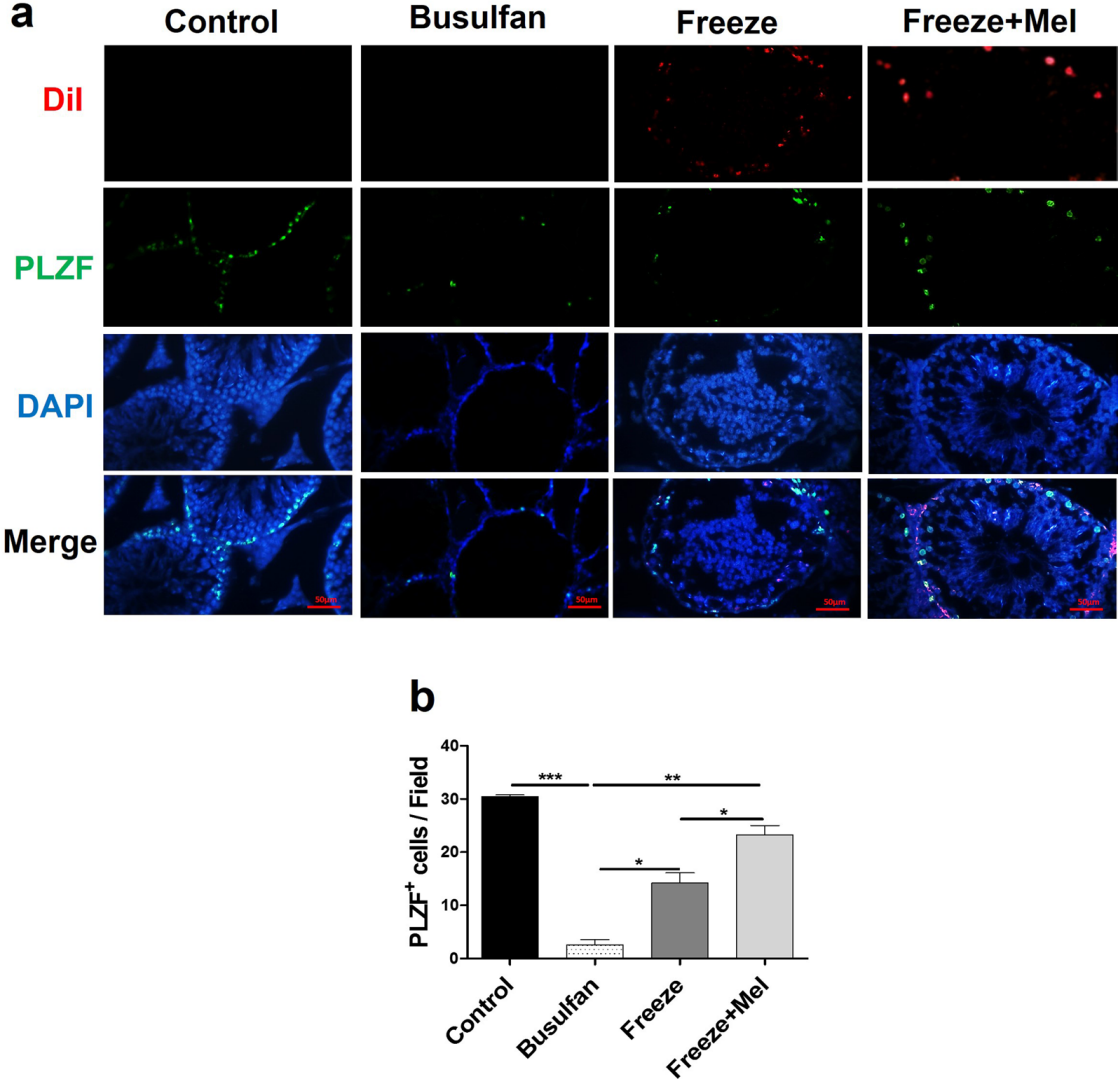
Immunofluorescence staining for identifying proliferation marker (PLZF) 8 weeks after transplantation of frozen–thawed SSCs into recipient testes. Post-thawed SSCs labeled with DiI (red) and nuclei are stained in the recipient testes by DAPI (blue). Green-stained SSCs represent the expression of PLZF marker in the control, Busulfan, Freeze and Freeze + Mel groups (**a**). The graph depicts the quantitative analysis for PLZF marker in different groups (**b**). Scale bars = 50 μm. The results are presented as mean ± SD. Significance is indicated by *p < 0.05; **p < 0.01; ***p < 0.001

**Fig. 6:**
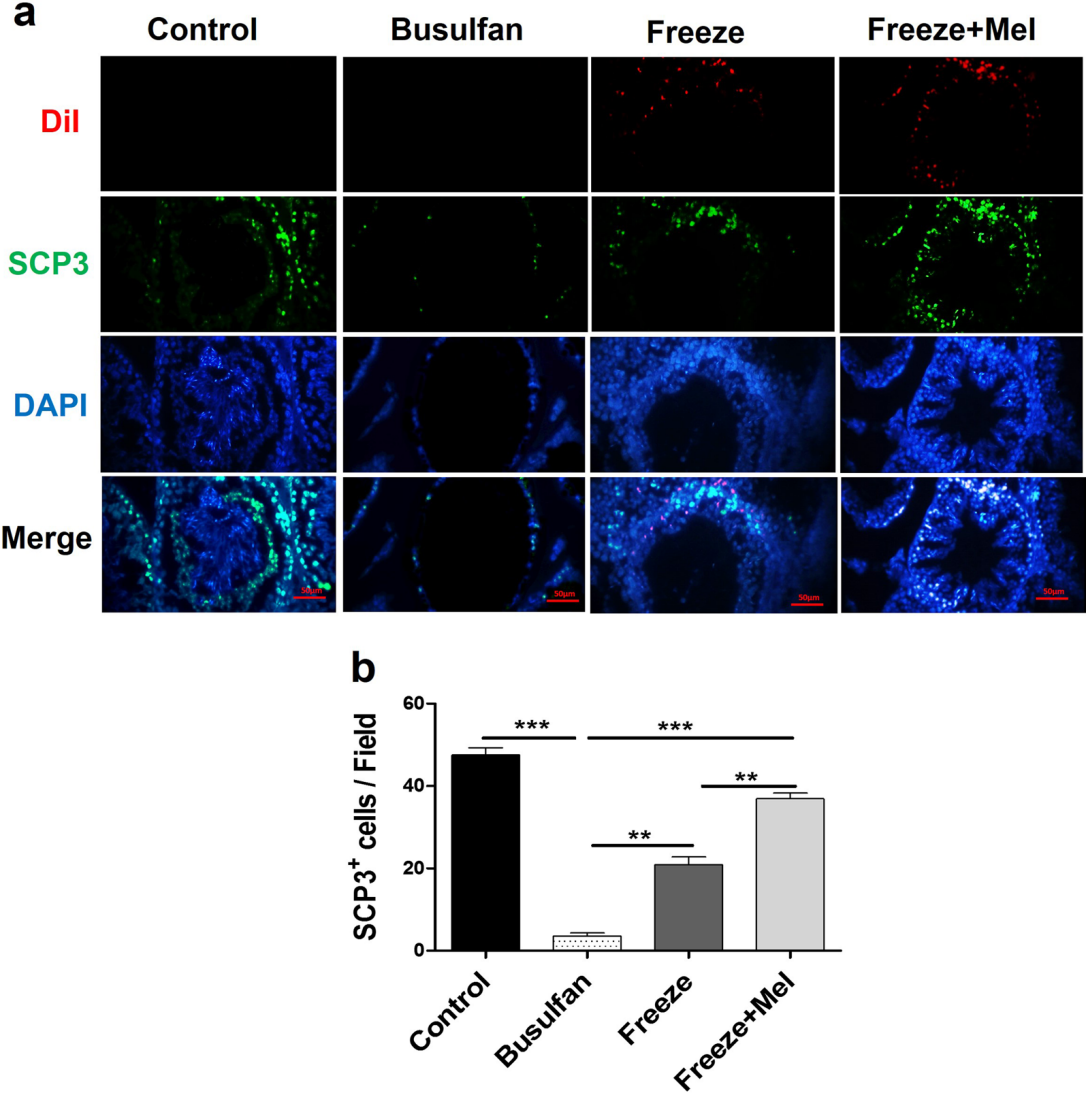
Immunofluorescence staining for identifying differentiation marker (SCP3) 8 weeks after transplantation of frozen–thawed SSCs into recipient testes. Post-thawed SSCs labeled with DiI (red) and nuclei are stained in the recipient testes by DAPI (blue). Green-stained SSCs represent the expression of SCP3 marker in the control, Busulfan, Freeze and Freeze + Mel groups (**a**). The graph depicts the quantitative analysis for SCP3 marker in different groups (**b**). Scale bars = 50 μm. The results are presented as mean ± SD. Significance is indicated by **p < 0.01; ***p < 0.001

Two months after transplantation, the expression rates of PLZF (proliferation) and SCP3 (differentiation) markers in SSCs transplanted into the recipient testes were evaluated using immunofluorescence. According to the results, the expression percentage of PLZF protein was 2.55 ± 1.48% (*p* < 0.001vs control), 14.20 ± 2.68% (*p* < 0.01 vs control) and 23.30 ± 2.40% (*p* > 0.05 vs control), in the busulfan, freeze and freeze + Mel groups, respectively (Fig. 5). The expression percentage of PLZF protein was significantly higher in the freeze + Mel group compared to the freeze group (*p* < 0.05) (Fig. 5). These results showed that freezing of germ cells reduced the expression of PLZF proliferation protein after transplantation. In addition, the results of quantitative analysis for expression of SCP3 protein showed that melatonin (freeze + Mel group) significantly increased the level of SCP3 differentiation protein compared to the freeze group (36.95 ± 1.90 vs 20.93 ± 2.65%) (*p* < 0.01) (Fig. 6). The results of SCP3 differentiation marker in the all group showed a pattern similar to the expression of PLZF proliferation marker. The high expression percentage of PLZF and SCP3 markers in the Freeze + Mel group compared to the Freeze group indicates the protective effect of melatonin on germ cells in the freezing medium before transplantation.

### Western blot findings for expression of GFRA1 and Stra8 proteins in SSCs

**Fig. 7:**
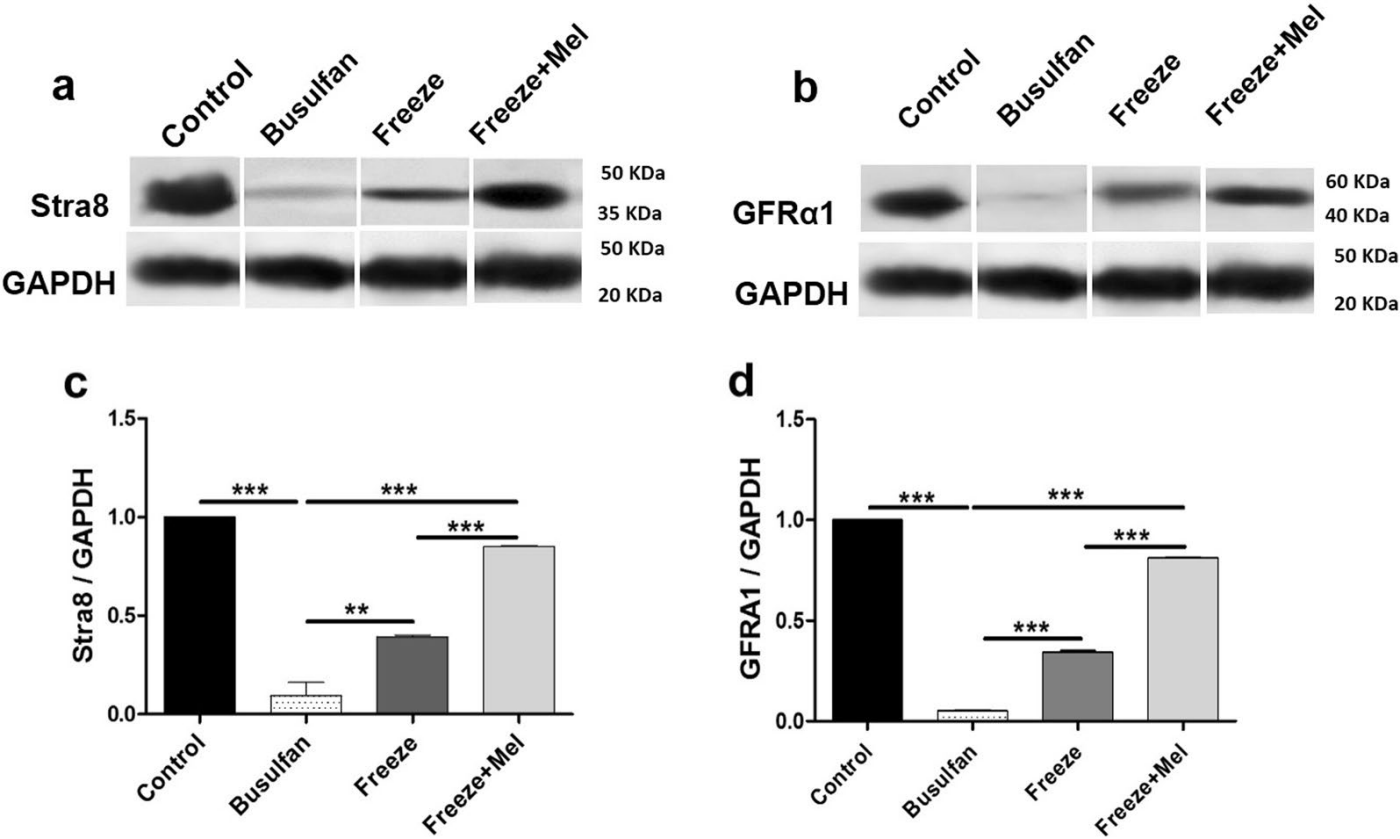
Western blot analysis for identifying proliferation (GFRa1) (**b**) and differentiation (Stra8) (**a**) markers 8 weeks after transplantation of frozen–thawed SSCs into recipient testes. GAPDH is used as an internal control. Graphs are indicative of the ratio for normalization of the density of the markers to the GAPDH (**c**, **d**). The results are presented as mean ± SD. Significance is indicated by **p ≤ 0.01; ***p < 0.001. The samples are derived from the same experiment and that gels/blots were processed in parallel (Additional file 1)

Two months after transplantation, recipient testes in all groups were assessed using western blotting for expression of GFRA1 (proliferation) and Stra8 (differentiation) proteins. According to the results of this experiment, the expression of GFRA1 protein increased significantly in the freeze + Mel (0.81 ± 0.06%) group compared to freeze (0.34 ± 0.03%) and busulfan (0.05 ± 0.01%) groups (*p* < 0.001) (Fig. 7b, d). Similarly, there was a significant increase in the Stra8 protein expression in the freeze + Mel group (0.85 ± 0.04) compared to freeze group (0.39 ± 0.02) and the busulfan group (0.09 ± 0.11) after transplantation (*p* < 0.001) (Fig. 7a, c). Therefore, increased expression of proliferation (GFRA1) and differentiation (Stra8) proteins in the freeze + Mel group compared to the freeze group during the 2 months after transplantation indicates that melatonin may have a protective effect on germ cells in the freezing medium during the freeze–thaw process.

### H&E staining findings

**Fig. 8:**
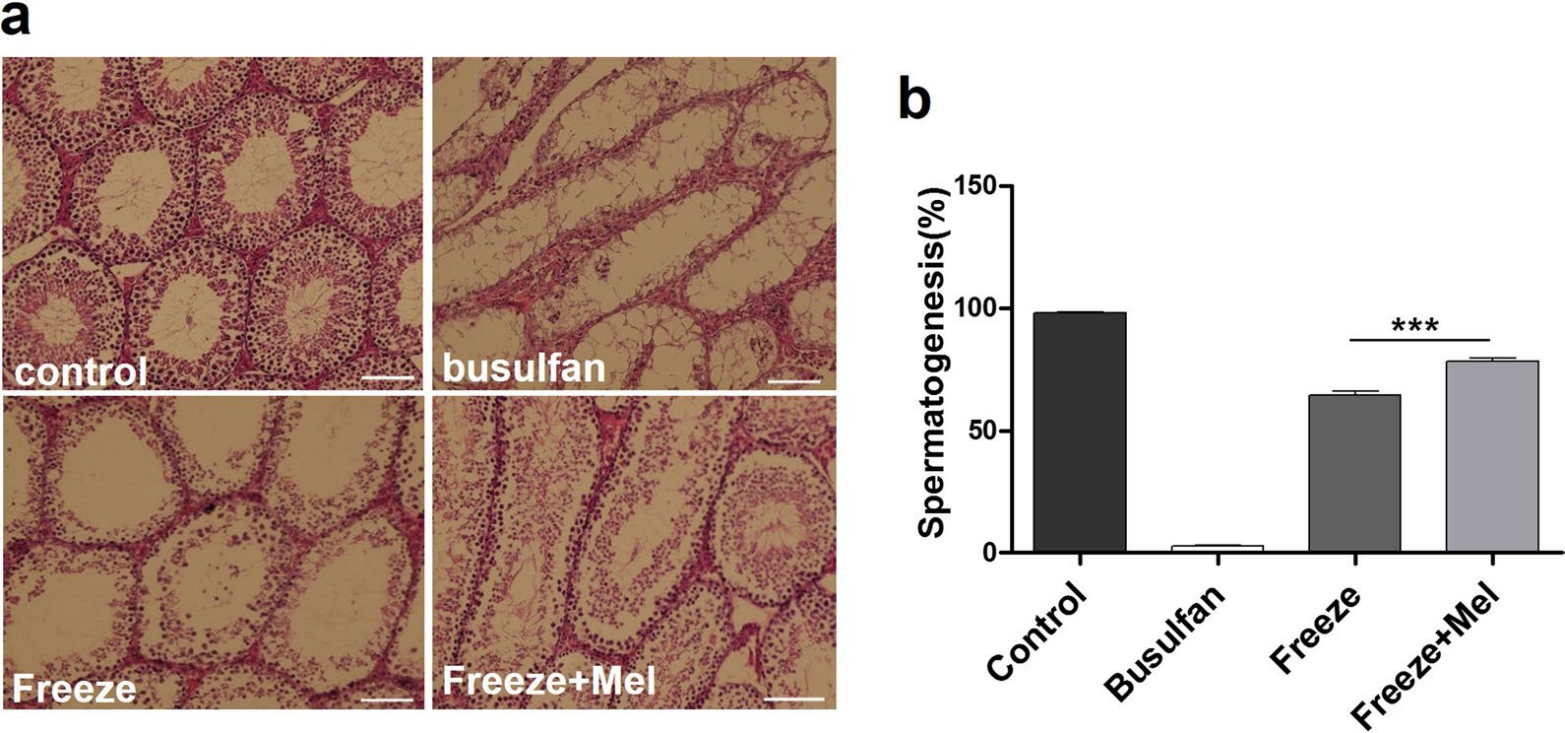
Hematoxylin and eosin staining of mouse testis after 2 months. Images show complete spermatogenesis in the control group, elimination of spermatogenesis in the busulfan group, and recovery of spermatogenesis after transplantation of frozen–thawed SSCs into the testes of azoospermic mice in freeze and freeze + Mel groups. The recovery of spermatogenesis was significant in the freeze + Mel group compared to freeze group. (**a**). Quantification of histological analyses indicates a significant increase in spermatogenesis recovery in freeze + Mel group compared to freeze group (**b**). Scale bar = 100 μm. The results are presented as mean ± SD. ***p < 0.001

Two months after transplantation of frozen–thawed SSCs, H&E staining was used to evaluate complete spermatogenesis in the tubules. Histological images of testicular sections in the control, busulfan, freeze (freezing of SSCs with basic freezing medium) and freeze + Mel (freezing of SSCs with melatonin) groups are presented in Fig. 8a. By counting the tubules, the recovery of complete spermatogenesis in the freeze + Mel group was significantly improved compared to the freeze group, and about 78% and 64% of the seminiferous tubules had the complete spermatogenesis for the freeze + Mel and freeze groups, respectively (Fig. 8b). The results showed that freezing of SSCs with melatonin improved the recovery of spermatogenesis following transplantation into the testis and protected SSCs from injuries caused by the freeze–thaw process.

## Discussion

With the development of treatment procedures and improving the survival of patients by cancer therapy drugs, the fertility of these patients is threatened in the future. Hence, researchers are trying to develop suitable methods to maintain fertility in people receiving anticancer and fertility-threatening drugs [33]. Freezing of SSCs isolated from immature testicles has been increasingly suggested as a method of preserving fertility before starting treatment for this population worldwide. The freezing protocol is associated with cell damage, which reduces the performance of SSCs after cryopreservation. Therefore, it is necessary to develop an optimal freezing technique to decrease the injury caused by the freezing–thawing process [34]. In the present study, we evaluated the protective effect of melatonin on cryopreservation of SSCs to establish an optimal cryopreservation protocol for preservation of SSCs. These results showed cryopreservation of SSCs by adding melatonin to the freezing medium improves the performance of SSCs (survival, proliferation and differentiation).

To identify SSCs, PLZF, Thy-1 and α-6 integrin markers were assessed by flow cytometry after enzymatic digestion and their isolation. The high expression for these three markers (PLZF, Thy-1 and α-6 integrin) in SSCs indicated the primary phases of the evolvement of the spermatogenic lineage in isolated SSCs whose differentiation process had not yet initiated and confirmed that SSCs collected after digestion enzymatic were in the undifferentiated stage. The high expression of SSC identification markers in this study was consistent with other studies [35–37].

Melatonin, as an antioxidant, anti-apoptotic and free-radical scavenger, is involved in cell protection [20]. In recent years, the protective effect of adding melatonin to the freezing medium has been tested on sperm viability after thawing [38–40]. The results demonstrated that melatonin at a concentration of 100 μM increased the viability of frozen–thawed SSCs. Therefore, a combination of 100 μM melatonin and the cryopreservation medium was considered as the optimal treatment in the present study. Similarly, Karimfar et al. found that adding melatonin at a concentration of 0.01 mM to the freezing medium increased post-thaw viability of cryopreserved human sperm [41]. Feng et al. also studied the protective effect of melatonin on the freezing–thawing process of goat SSCs and suggested that melatonin improved the viability of post-thawed SSCs, which confirms the results of the present study [42].

Oxidative stress in cells occurs when there is an imbalance between oxidants and antioxidants due to the generation of super-physiological levels of reactive oxygen species (ROS). The very low temperature in the cryopreservation method causes oxidative stress in the cells [43]. Reactive oxygen species (ROS) such as hydrogen peroxide (H2O2), superoxide anion radical (O^·−2^) and hydroxyl radical (·OH) are produced in cells during cryopreservation [44]. Overproduction of reactive oxygen species beyond normal physiological cellular levels during cryopreservation can contribute to injuries such as lipid peroxidation [45], protein oxidation [46], DNA damage [47], and even apoptosis [8] in cells. During the freezing–thawing process, the addition of antioxidants to the freezing medium containing cryoprotectant agents (CPAs) helps to reduce ROS generation [48], clear intracellular ROS [8] and reduce oxidative stress [49] in reproductive cells. Recent studies have shown that melatonin as a potent antioxidant has the capacity to eliminate the toxic effects of ROS and neutralize free radicals in the cryopreservation of the sperm of various species of animals and humans [39, 50, 51]. The results of the present study confirmed that the cryopreservation procedure led to the accumulation of intracellular ROS in thawed SSCs during the freezing–thawing process. This increase in the ROS level is significantly reduced by adding melatonin to the cryopreservation medium containing SSCs. In line with our results, Najafi et al. found that sperm cryopreservation was associated with enhanced ROS levels, and treatment of freezing medium with melatonin as an antioxidant reduced ROS levels after thawing [52]. Furthermore, Feng et al. found that melatonin had a protective effect on cryopreserved SSCs, and treatment of goat SSCs with melatonin reduced intracellular ROS production during cryopreservation [42].

In addition to reducing the production of ROS levels in cells, melatonin is effective in regulating the signaling pathway of apoptosis by adjusting the expression ratio of apoptosis-related markers, including Bax and Bcl2 [53]. In this study, immunocytochemistry was used to measure the expression of apoptosis-related proteins in SSCs after freezing and thawing. The results showed that adding melatonin to the cryopreservation medium reduced apoptosis in SSCs by reducing the expression of Bax (pro-apoptotic) protein and increasing the expression of Bcl2 (anti-apoptotic) protein. This finding is consistent with the results of a study by Khan et al. who examined the effect of melatonin after induced apoptosis in mice testes and suggested that melatonin acted as a potent anti-apoptotic by reducing Bax expression and increasing Bcl2 expression [54]. Similarly, Goe et al. found that melatonin increased the expression of Bcl2 as an anti-apoptotic marker and decreased the expression of Bax as a pro-apoptotic marker and could protect against stress-induced testicular cell apoptosis in mice [55].

Melatonin, with two different receptor-dependent and receptor-independent pathways, is effectively involved in regulation of many biological activities of the reproductive system. Due to the presence of melatonin membrane receptors (MT1 and MT2) in reproductive cells, many studies have investigated the effect of melatonin via the membrane receptor-dependent pathway in the ovary and testis [56, 57]. Melatonin and its metabolites are directly involved in the elimination of free radicals and the neutralization of highly toxic oxidizing agents including peroxynitrite anion (ONOO–) and hydroxyl radical (·OH) through a receptor-independent pathway [20]. In general, the mechanism of action of melatonin in the receptor-independent pathway includes prevention of reactive oxygen species (ROS) formation, increasing the actuality of antioxidant enzymes, and protecting cells from ROS-induced damage [58]. Therefore, we believe that melatonin as a cryoprotective agent has the ability to protect SSCs during freezing and thawing by reducing Bax expression, increasing Bcl2 expression, preventing the formation of intracellular ROS and increasing viability. Although the exact mechanism of the effect of melatonin in reducing the damage caused by cryopreservation is unknown, two possibilities are suggested for the protection of frozen–thawed SSCs with melatonin: (1) In the receptor-independent pathway, melatonin may directly help to protect SSCs by clearing ROS and scavenging free radicals, and (2) melatonin, by forming a sheath around cells, may minimize cell dehydration or alter the physical state of the cryopreservation medium. Melatonin in the cell culture medium not only increases the survival and proliferation of SSCs but also is effective in differentiating SSCs [16, 59]. In the present study, the effect of a cryopreservation medium containing melatonin on the biological function of SSCs (proliferation and differentiation) was evaluated 2 months after transplantation of thawed SSCs into mice testes.

GDNF family receptor α-1 (GFRA1) and promyelocytic leukemia zinc finger (PLZF) markers are expressed in undifferentiated spermatogonia (As-Aal). GFRA1 regulates self-renewal in undifferentiated SSCs and prevents the differentiation process of SSCs [60, 61]. PLZF is essential for the proliferation and self-renewal of SSCs and plays a key role in maintaining the undifferentiated state of SSCs in seminiferous tubules [62].

The present study found that the expression percentage of PLZF and GFRα1 proliferation proteins markedly decreased in the busulfan group and increased in the transplantation groups (freeze and freeze + Mel groups). Among the transplantation groups, the increase in the expression of PLZF and GFRα1 markers was notable in the cryopreservation with 100 μM melatonin compared to the cryopreservation with basic freezing medium. Increased expression of PLZF and GFRα1 proteins in transplantation groups indicated an increase in the number and proliferation of thawed SSCs after transplantation into azoospermic testes. These results confirmed other findings of this study and indicated that adding melatonin to the cryopreservation medium reduced the cryoinjuries caused by the freezing–thawing process and protected the SSCs during cryopreservation. Similarly, Jung et al. found that adding antioxidant supplements to the culture medium of murine SSCs increased the expression of PLZF and GFRα1 proteins (undifferentiated markers for spermatogonia) compared to SSCs without antioxidant supplementation after the freezing–thawing process and improved post-thaw recovery of murine cryopreserved SSCs via preventing ROS production and apoptosis [63]. Azizollahi et al. reported that significant expression of proliferation markers (PLZF and GFRα1) in SSCs after transplantation into the testes of azoospermic mice indicated an increase in the number and proliferation of transplanted SSCs [64].

Stimulated by retinoic acid gene 8 (Stra8) and synaptonemal complex protein 3 (SCP3) have been identified as meiosis-related markers. In mammalian germ cells, Stra8 is expressed as a specific gene during transition from mitosis to meiosis, which plays an important role in initiating the phase of meiosis in these cells during spermatogenesis. SCP3 is essential for meiosis and differentiation of germ cells [65, 66]. The results of the present study showed a marked increase in the expression of differentiation proteins (Stra8 and SCP3) after transplantation of frozen–thawed SSCs into busulfan-induced azoospermic mice. Increased expression of Stra8 and SCP3 was considerable in the freeze + Mel group compared to the freeze group. Malekzadeh et al. found that adding pentoxifylline to the freezing medium improved the recovery of post-thaw SSCs and increased the expression of proliferation (ID4 and Ddx4) and differentiation (SCP3 and C-Kit) proteins compared to frozen–thawed SSCs in the basic freezing medium after transplantation into azoospermic mice [37].

The researchers have shown that cryopreservation and transplantation of frozen–thawed SSCs into infertile mice can help to restore spermatogenesis and treatment fertility [67, 68]. We found that spermatogenesis was successfully restored from frozen–thawed SSCs in the recipient testes; however, the results showed better recovery of spermatogenesis in cryopreserved SSCs with melatonin (freeze + Mel group) compared to cryopreserved SSCs with basic medium (freeze group). In keeping with our study, Ali Akbari et al. studied the effect of adding antioxidant supplements to a basic freezing medium containing SSCs and suggested that cryopreservation of SSCs with antioxidant supplements not only improved the biological function of SSCs but also was effective in restoring spermatogenesis from post-thawed SSCs after transplantation [32]. Enhancement of spermatogenesis in cryopreserved SSCs with melatonin confirms that adding melatonin to CPAs (i.e., DMSO) improves the recovery of post-thaw SSCs and can provide the possibility of long-term storage of SSCs in prepubertal boys who have malignant illnesses and are waiting for radiotherapy and chemotherapy. In summary, the results of our study showed that adding melatonin to the cryopreservation media markedly increased the viability and reduced intracellular ROS generation and apoptosis (by decreasing BAX and increasing BCL2) in the frozen–thawed SSCs before transplantation into recipient testes. Also, our findings after transplantation of frozen–thawed cells with melatonin confirmed the protective role of melatonin in cryopreservation media. The expression levels of proliferation (PLZF and GFRα1) and differentiation (Stra8 and SCP3) proteins and resumption of spermatogenesis from frozen–thawed SSCs in the freezing media containing melatonin group was significantly increased compared to the basic freezing media group. Therefore, the present study showed that freezing and long-term storage of SSCs with melatonin and then their transplantation after thawing can be a suitable option for fertility preservation of infertile men in the future.

## Conclusions

The findings showed that adding 100 μM melatonin to a freezing medium containing a cryoprotectant agent (DMSO) protected SSCs by preventing intracellular ROS production and inhibiting apoptosis and increased the survival rate and recovery of SSCs after thawing. Therefore, a combination of the melatonin and cryoprotectant agents (CPAs), as a novel approach in cryopreservation, can improve the proliferation, differentiation, and recovery of spermatogenesis from frozen–thawed SSCs in the recipient testes by reducing the damage caused during cryopreservation. This new approach to cryopreservation, i.e., adding melatonin to CPAs, could be a promising strategy in cryopreservation and long-term storage of SSCs to maintain fertility in prepubertal boys who suffer from cancer.

## Supplementary Information

**Additional file 1.** Original western blot bands.

## Data Availability

The datasets used and/or analyzed during the current study are available from the corresponding author on reasonable request.

## Abbreviations

SSCs: Spermatogonial stem cells
CPAs: Cryoprotectant agents
DMSO: Dimethyl sulfoxide
EG: Ethylene glycol
ROS: Reactive oxygen species
DMEM: Dulbecco’s modified Eagle medium
FITC: Fluorescein isothiocyanate
BFGF: Basic fibroblast growth factor
OD: Optical density
BSA: Bovine serum albumin
HRP: Horse radish peroxidase
