# Supplementary figures and images for: Melatonin in cryopreservation media improves transplantation efficiency of frozen–thawed spermatogonial stem cells into testes of azoospermic mice
Source: Stem Cell Res Ther. 2022 Jul 26;13:346. doi: 10.1186/s13287-022-03029-1 (PMC9327150; doi:10.1186/s13287-022-03029-1)

**Stra8**

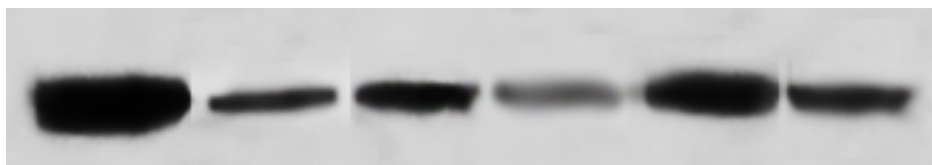

**Stra8**

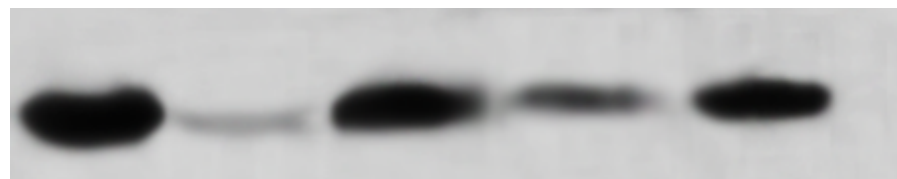

*50KDa*  
*35KDa*

**GFRa1**

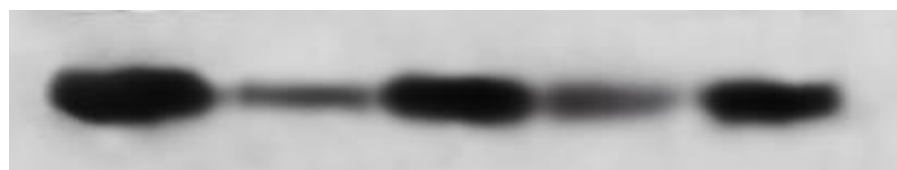

*60KDa*  
*40KDa*

**GAPDH**

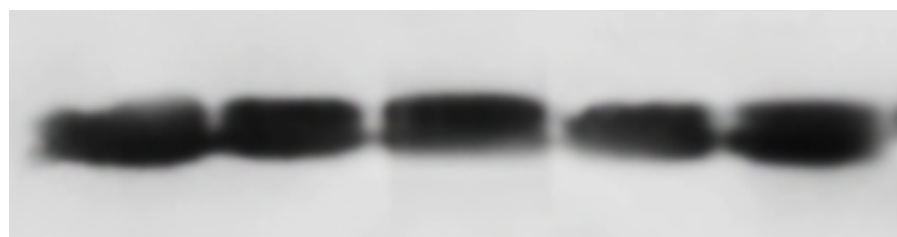

*50KDa*

*20KDa*

**GFRa1**

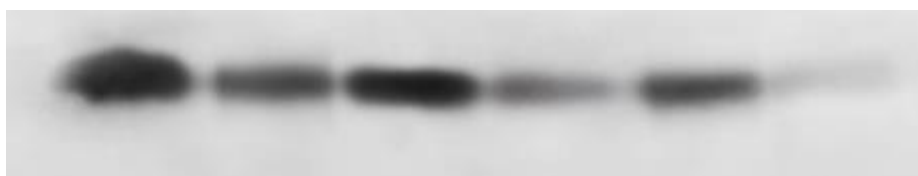

**Stra8**

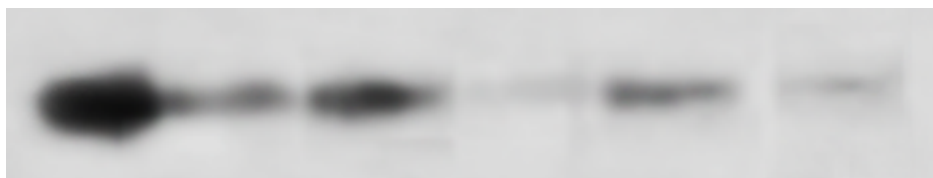

**GFRa1**

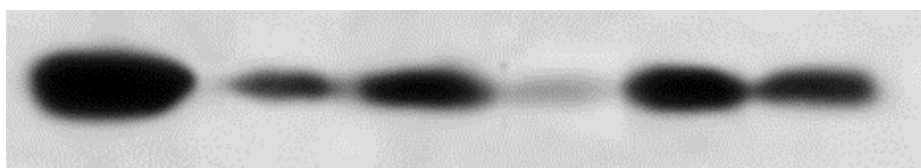

Supplement: Supplementary file 1 — Additional file 1. Original western blot bands. [file 13287_2022_3029_MOESM1_ESM.pdf]
